# Dataset on lipid profile of bovine oocytes exposed to Lα-phosphatidylcholine during in vitro maturation investigated by MALDI mass spectrometry and gas chromatography-flame ionization detection

**DOI:** 10.1016/j.dib.2017.06.026

**Published:** 2017-06-17

**Authors:** Alessandra A. Vireque, Christina R. Ferreira, Rafael R. Hatanaka, Alessandra Tata, Katia Roberta A. Belaz, Vanessa G. Santos, Marcos N. Eberlin, Marcos Felipe Silva de Sá, Rui A. Ferriani, Ana Carolina J.S. Rosa e Silva

**Affiliations:** a Department of Obstetrics and Gynecology, Ribeirão Preto Medical School, University of São Paulo, 14049‐900, Ribeirão Preto, SP Brazil; b ThoMSon Mass Spectrometry Laboratory, Institute of Chemistry, University of Campinas, 13083-970 Campinas, SP, Brazil; c Center for Monitoring and Research of the Quality of Fuels, Biofuels, Crude Oil and Derivatives–CEMPEQC, Institute of Chemistry, UNESP–São Paulo State University, 14800-900 Araraquara, SP, Brazil

**Keywords:** Phospholipid, Fatty acids, Oocyte maturation, MALDI-MS, GC-FID

## Abstract

Data presented in this article are related with the research article entitled “Effect of soybean phosphatidylcholine on lipid profile of bovine oocytes matured in vitro” [1]. This article describes the differences in the relative abundance of the lipid ions detected by matrix-assisted laser desorption/ionization mass spectrometry (MALDI-MS) in control and Lα-phosphatidylcholine-treated oocytes. In addition, the fatty acids (FA) content in pure Lα-phosphatidylcholine supplement and oocytes was analyzed by gas chromatography-flame ionization detection (GC-FID). The dataset provides information and inputs for further studies aiming to optimize *in vitro* maturation conditions and cryotolerance of mammalian oocytes.

**Specifications Table**

**Table:** 

Subject area	*Biology*
More specific subject area	*Assisted reproductive technology*
Type of data	*Text file, table, figures*
How data was acquired	*Autoflex III MALDI time-of-flight mass spectrometer (Bruker Daltonics, Bremen, Germany) and GC-2010 capillary gas chromatograph system (Shimadzu, Tokyo, Japan)*
Data format	*Analyzed*
Experimental factors	*NA*
Experimental features	*Phospholipid and fatty acid detection by MALTI-TOF mass spectrometry and GC-FID in oocytes*
Data source location	*NA*
Data accessibility	*The data are available with this article*

**Value of the data**

• The data presents a comparison of the relative abundance of lipid ions detected by MALDI-MS in sampled control oocytes and oocytes supplemented with Lα-phosphatidylcholine at 50 and 100 μM during in vitro maturation.
• First FA content analysis by GC-FID of oocytes exposed to phosphatidylcholines during in vitro maturation.
• The samples were subjected to transmethylation/methylation procedures according to ISO 12966-2:2011 standard [2] and FAME (fatty acid methyl esters) were analyzed under conditions described in ISO 12966-4:2015 standard [3] and could be compared to others protocols.
• This data allow other researchers to develop targeted strategies for studying the effects of phospholipids on *in vitro* maturation of oocytes, embryo culture and cryopreservation.

## 1 Data

**Fig. 1:**
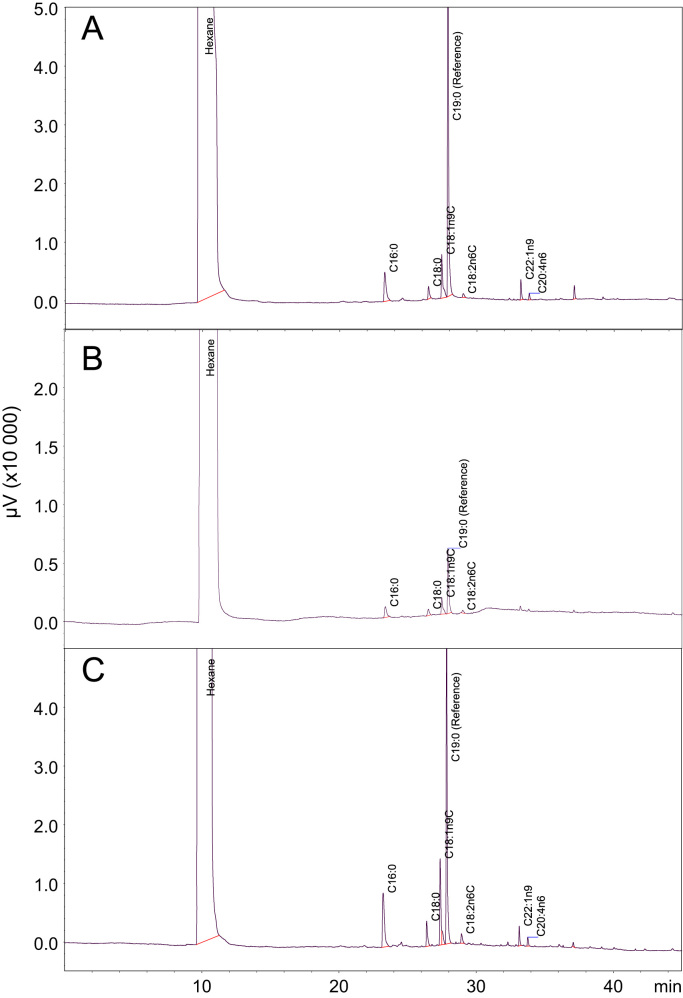
Identification chromatograms of bovine oocytes exposed to 100 μM Lα-phosphatidylcholine during in vitro maturation and analyzed by GC-FID method. Immature (fresh) oocytes (A); control in vitro-matured oocytes (B) and PC-supplemented oocytes (C).

**Fig. 2:**
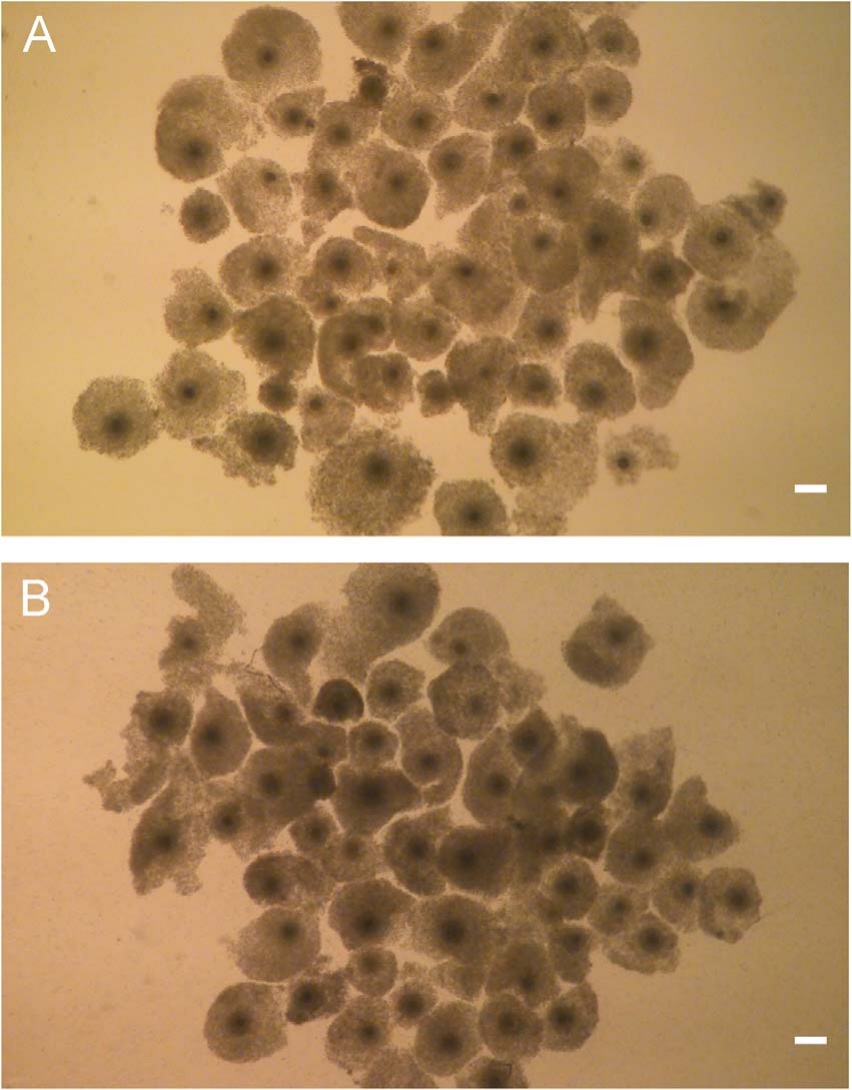
Bovine cumulus-oocyte complexes matured *in vitro* in TCM medium supplemented with 10% fetal bovine serum. Control oocytes (A); oocytes supplemented with Lα-phosphatidylcholine at 100 μM (B). Bar = 100 μm.

**Fig. 3:**
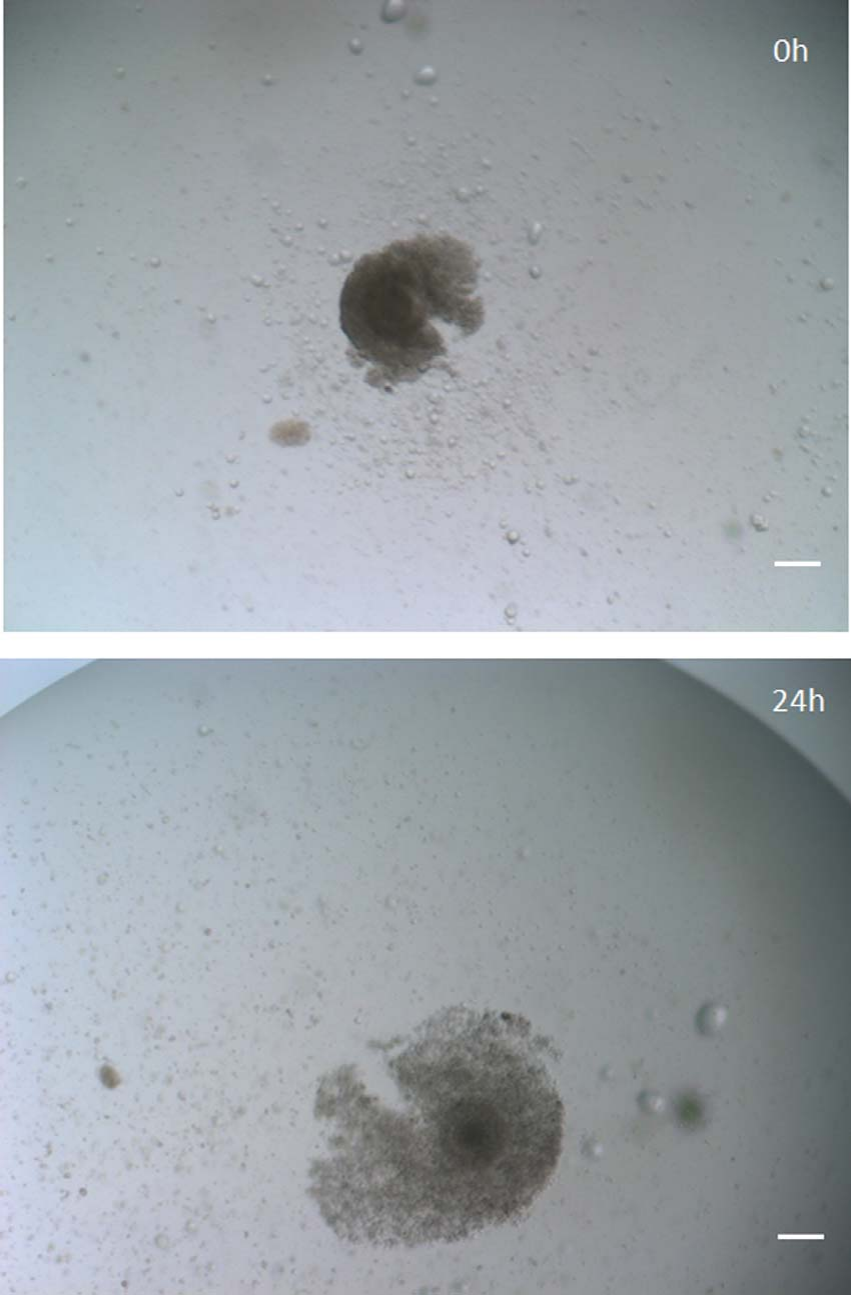
Cumulus oophorus expansion of bovine matured *in vitro* in TCM-PC100. Bar = 100 µm.

**Fig. 4:**
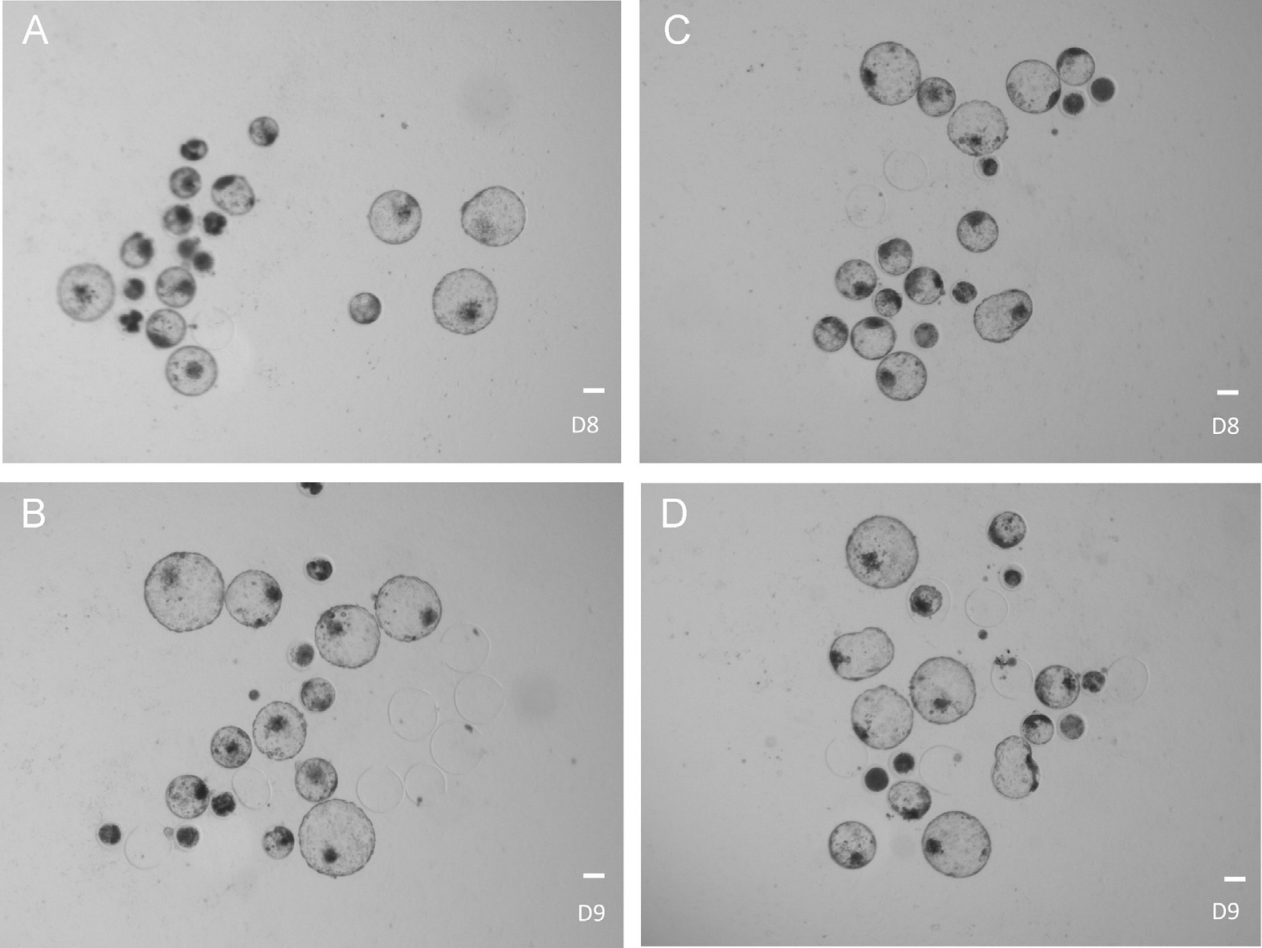
Bovine embryos produced *in vitro* from oocytes exposed to Lα-phosphatidylcholine during IVM. A-B: control embryos derived from oocytes unexposed to PC supplement, C-D: embryos derived from PC-treated oocytes; D8/D9: days of embryo culture post-insemination. Bar = 100 μm.

The dataset of this article provides information on lipid profile and FA content in bovine oocytes supplemented with Lα-phosphatidylcholine (PC) during in vitro maturation (IVM). The Figs. 1 shows chromatograms of FA detected by GC-FID in control and PC-supplemented oocytes. The Fig. 1, Fig. 2, Fig. 3, Fig. 4 are optical images showing the morphology of PC oocytes during IVM and resulting in vitro produced embryos. Table 1 contains a list of all significant ions detected by MALDI-MS in tissue culture medium (TCM) and bovine oocytes. Table 2 shows the differences in the relative abundance of the five significant lipid ions detected in oocytes matured in TCM medium supplemented with purified soybean PC at 50 or 100 µM [1]. Table 3 contains a list of FA quantified in Lα-phosphatidylcholine supplement.

**Table 1:** Phospholipids (PL) and triacylglycerols (TAG) identified via MALDI(+)-MS in TCM medium and bovine oocytes supplemented with Lα-phosphatidylcholine at 50 and 100 µM during in vitro maturation.

*m/z*	*Lipid ion (carbons:unsaturation)*
725.5	[SM (16:0) + Na]^+^
734.6	[PC (32:0) + H]^+^
758.6	[PC (34:2) + H]^+^
782.6	[PC (36:4) + H]+, [PC (34:1) + Na]^+^
804.6	[PC(38:7) + H]+, [PC(36:4)+ Na]^+^
806.6	[PC (38:6) + H]^+^, [PC(36:3)+ Na]^+^

Identification based on MALDI-MS lipid profile studies as described by Ferreira et al. [4] as well as two lipid databases (http://lipidsearch.jp and http://www.lipidmaps.org).

**Table 2:** Averaged relative intensity of PL detected by MALDI-MS in oocytes supplemented with soybean phosphatidylcholine (PC) at 50 and 100 µM during in vitro maturation in TCM medium.

***m/z***	**TCM**	**TCM PC 50**	**TCM PC 100**
**725.5**	2	4	38
**734.6**	10	18	42
**758.6**	5	15	48
**782.6**	10	40	80
**806.6**	0	2	10

**Table 3:** Fatty acid content in Lα-phosphatidylcholine supplement analyzed by gas chromatography-flame ionization detection (GC-FID) method.

**Name**	**Fatty acid**	**% of Área**
**Palmitic acid**	C16:0	42,62
**Stearic acid**	C18:0	33,93
**Oleic acid**	C18:1	1,10
**Linoleic acid**	C18:2	18,83
**Linolenic acid**	C18:3	2,41
**Heneicosanoic acid**	C21:0	1,11

## 2 Experimental design, materials and methods

The Lα-phosphatidylcholine was solubilized in pure DMSO and then diluted in TCM medium [5] supplemented with 0.05% bovine BSA (bovine serum albumin) to give a 10 mM stock solution (1:1, by vol) and stored at -20 °C in sterile vials sealed under nitrogen. On the day of use, the stock solution was diluted in culture medium at 50 or 100 µM. Pools of oocytes from each group (TCM, TCM-PC50 and TCM-PC100) were matured, prepared and analyzed by MALDI-MS as previously described [1].

For FA identification and quantitation by GC-FID, the PC sample or oocytes were subjected to transmethylation/methylation procedures under sequential alkaline and acid conditions, according to ISO 12966-2:2011 standard [2]. To the each oocyte sample, n = 200–400 per group [6], suspended in 0.1 mL PBS were added 30 μl C19 standard (concentration of 0.5 mg/g) and 100 µL 0.2 M NaOH methanolic solution. The samples were placed in a water bath at 80 °C for 40 min and shaken manually every 5 minutes. In the next step, 100 μL of 1.0 M methanolic solution of H_2_SO_4_ was added and the samples were placed additionally in a water bath at 80 °C. Then 200 μL of aqueous NaCl solution and 100 μL of hexane were added and the vial was shaken vigorously. The upper phase containing hexane and FAME was then carefully collected and transferred to another vial. The extraction procedure was repeated two more times with 100 μl of hexane and under vigorous stirring for FAME extraction. FAME were analyzed by capillary gas chromatography, under conditions described in ISO 12966-4:2015 standard [3]. Peaks were identified by comparison with a commercial Certified Reference Material FAME mix C4:0 to C24:0 purchased from Supelco Inc. (Bellefonte, PA) and quantified by area normalization (Table 3).
